# Specific Treatment Exists for SARS-CoV-2 ARDS

**DOI:** 10.3390/vaccines9060635

**Published:** 2021-06-10

**Authors:** Badar Kanwar, Chul Joong Lee, Jong-Hoon Lee

**Affiliations:** 1 Department of Pulmonary Critical Care Medicine, Hunt Regional Hospital, Greenville, TX 75401, USA; Bkanwar2@yahoo.com; 2 Zein Pain Clinic Seongbuk, Seoul 02830, Korea; cjlee.mdphd@gmail.com; 3 Science & Research Center, Seoul National University College of Medicine, Seoul 03080, Korea

**Keywords:** brainstem, inflammasome, oral DNA vaccine

## Abstract

The COVID-19 pandemic, caused by severe acute respiratory syndrome coronavirus 2 (SARS-CoV-2), seems to be difficult to overcome. A pandemic of such a scale has not been seen since the 1918 influenza pandemic. Although the predominant clinical presentation is respiratory disease, neurological manifestations and sequelae are increasingly being recognized. We observed a case series of rapid recovery of ARDS within 24 h in the preliminary clinical features of COVID-19 ARDS-associated neurological disease. It was also noted that by 15 April, 2021, there was no SARS-CoV-2 ARDS on Sorok Island in South Korea, where lepers had been living together. We compared each of dapsone’s effects on humans and considered those of SARS-CoV-2. Dapsone showed different effects in the brain. The Sorokdo National Hospital reported a relationship between dapsone and the neuroinflammasome of Alzheimer’s disease (AD) in Sorok Island from January 2005 to June 2020. AD prevalence was low in the leprosy patient group who took dapsone regularly. The preliminary cross-sectional study of the trial group (22 subjects) and the control group (22 subjects) in the Hunt Regional Hospital reported the following results: The chi-square statistic is 5.1836. The p-value is 0.022801. The result is considered significant at *p* < 0.05. The results from the medical treatment from 21 December to 29 December 2020 were considered. The mortality rates at the ARDS onset stage were 0% with dapsone administered as a standard COVID-19 treatment and 40% without dapsone administered as a standard COVID-19 treatment, respectively. Based on the respiratory failure and sudden high death rate originating from the involvement of the brainstem, especially the pre-Bötzinger complex, dapsone can be used to significantly reduce the incidence of the cases of acute respiratory distress syndrome and other illnesses caused by SARS-CoV-2.

## 1. Introduction

In December 2019, there was a local outbreak of pneumonia of unknown cause in Wuhan (Hubei, China). This precipitated the COVID-19 pandemic, caused by the novel coronavirus severe acute respiratory syndrome coronavirus 2 (SARS-CoV-2), which has resulted in more than 145,289,885 confirmed cases globally and more than 3,083,440 deaths [1,2]. Established public health measures for the control of SARS-CoV-2 continue to rely on social distancing; frequent hand washing; face coverings; and practical test, trace, and isolate systems [3].

The COVID-19 pandemic has led to the reconsideration of public health policies, healthcare providers, and the virus’ effects on the central nervous system. During the period of the pandemic, only one researcher found a way to treat the large number of patients who suffered from neuroinflammation or neuroinflammasome as a result of SARS-CoV-2 [4,5,6]. He explained that the respiratory failure and sudden high death rate in COVID-19 acute respiratory distress syndrome (ARDS) patients originated from the involvement of the brainstem, especially the pre-Bötzinger complex [7,8]. His team used dapsone in COVID-19 ARDS patients to compete with SARS-CoV-2 inflammasomes.

## 2. Evidence from Brain Diseases

**Figure 1:**
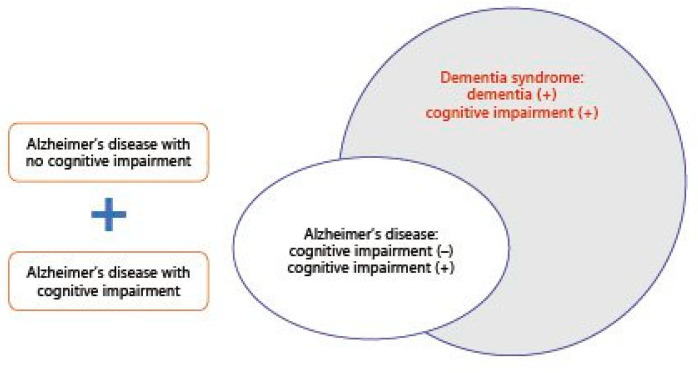
Scope of Alzheimer’s disease and dementia syndrome [9].

A study on the elderly diagnosed with mild cognitive impairment (MCI) from 2008 to 3 January 2021 was designed to classify MCI and Alzheimer’s disease (AD) cases as dementia syndrome with(out) cognitive impairment (Figure 1).

**Figure 2:**
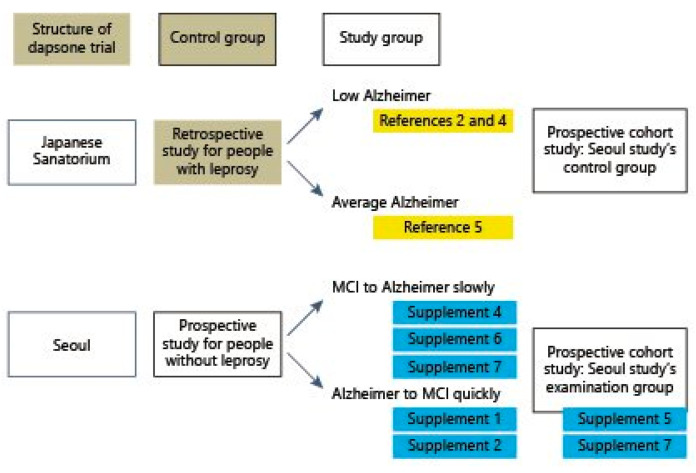
Structure and result of prospective cohort.

And it pondered once more the Japanese null hypothesis for dapsone’s therapeutic potential for AD [10,11,12] (Figure 2).

The AD patients recovered to MCI states, and their quality of daily life was improved, with the use of dapsone [9,13].

The authors of this study also created and expanded the Seoul cohort based on the thesis that dapsone’s sulfone group would protect cerebrovascular blood vessels through the action of the sulfone amide group of dapsone, and they conducted research on AD and Parkinson’s disease (PD) patients [9]. In December 2020, the study confirmed that its therapeutic mechanism was as a neuroinflammasome competitor [6,13,14].

Before dapsone was identified as an inflammasome competitor, clinicians used it as an adjuvant [15], alternative [16], augmentation [17,18], or active ingredient [19,20]. Dapsone has been used as a therapeutic agent for mild cognitive impairment [9,21], AD [14], PD [22,23], seizures [24], strokes [13,25,26], and finally, COVID-19 ARDS [6,27].

## 3. Projected Neurological Findings for COVID-19-Associated ARDS

He suggested that dapsone might be a treatment for SARS-CoV-2 in January 2020 because of the clinical manifestations of SARS-CoV-2 and dapsone’s effects in humans. He compared each of dapsone’s effects on humans and considered those of SARS-CoV-2 [6]. However, dapsone showed different effects in the brain (Table 1).

**Figure 3:**
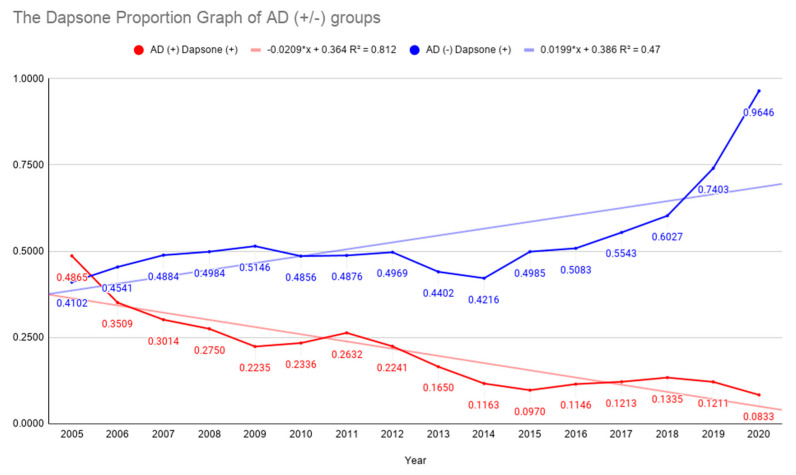
Dapsone proportion graph with(out) Alzheimer’s disease groups. This study was conducted from 2005 to 2020 based on the medical records of the Sorokdo National Hospital that was established in May 1916 to treat leprosy [9]. However, the patient research in the report was initiated by Sister Marianne Stoeger and Sister Margaritha Pissarek, who moved to Sorok Island in February 1962 and October 1967, respectively. They left Sorok Island on 21 November 2005, returning to their homelands. The leprosy patients here have taken dapsone all their lives. The AD prevalence rate was very low in the leprosy patient group who took dapsone regularly.

South Korea has suffered severely from SARS-CoV (2002), influenza A virus subtype H1N1 (2009), MERS (2015), and SARS-CoV-2 (2020), and has reported the recurrence of leprosy according to regular medical examinations and diagnosis. The annual recurrence rate for leprosy has been below 0.02 (%) for the past ten years. The Korea Centers for Disease Control and Prevention (KCDC) are supplying dapsone to leprosy patients. The Sorokdo National Hospital is responsible for the treatment of respiratory infectious diseases and has reported no prevalence of each pandemic of SARS-CoV (2002), influenza A virus subtype H1N1 (2009), MERS (2015), or SARS-CoV-2 (2020) [6]. The Sorokdo National Hospital reported a relationship between dapsone and AD in Sorok Island from January 2005 to June 2020. AD prevalence is low in the leprosy patient group who took dapsone regularly (Table 2) (Figure 3).

CNS diseases are caused by inflammation and oxidative stress. The efficiency with which drugs acting on the CNS traverse the blood–brain barrier (BBB) is important [45]. Dapsone appears to have more significant anti-inflammatory effects due to its effective accumulation in the CSF in humans [46]. Dapsone’s action as a neuroinflammasome competitor [6,13] can be conjectured by comparing the AD prevalence in leprosy patients who have taken dapsone with that in those who have not [14]. Dapsone was deemed a neuroinflammasome competitor in conclusion.

In accordance with the Information Disclosure Act, the National Sorokdo Hospital responded as follows in the response form to the request for information disclosure on 15 April 2021—7767726:

“Regarding the “COVID-19 infection status among Hansen people of Sorokdo National Hospital” requested by the claimant, we will respond as follows: We are pleased to inform you that there are no patients infected with COVID-19 among Hansen Disease patients at National Sorokdo Hospital”.

## 4. The Preliminary Clinical Features of Neurological Disease Associated with COVID-19 ARDS

Dr. B.K. insisted on using dapsone for ARDS patients as an inflammasome competitor at a COVID-19 committee at the Hunt Regional Medical Center in the U.S. The committee accepted the off-label use of dapsone with written informed consent after explaining its side effects and monitored its clinical results in terms of cytokine downregulation [47].

Dapsone treatment was commenced in a COVID-19 patient with progressively worsening hypoxia at the request of the family. Dapsone resulted in unexpectedly rapid clinical improvement within 24 h (Supplementary Materials Section 2; Case Reports, Case 3) (Table 3).

We used dapsone in 43 patients: 19 patients in the first period, 22 in the second period, and 2 (Dr. B.K.’s family) in the third period. We established objective criteria for improvement, including a reduction in FIO_2_ requirement and decrease in the progression of hypoxia. ARDS onset cases were based on FIO_2_ needs via simple nasal cannulation of up to 15 L/min. The criteria for aggravated cases were FIO_2_ administered via an HFNC (high-flow nasal cannula) of 95–100% or BiPAP (bilevel positive airway pressure). The criteria for severe cases of ARDS was the need for mechanical ventilation.

In the Hunt Regional Hospital intensive care unit (ICU), the medical staff in charge of COVID-19 patients changed approximately every eight days. The COVID-19 committee at the Hunt Regional Medical Center re-evaluated the results for two weeks after the first-period trial. Dr B.K. was ordered to stop prescribing dapsone. Following the committee’s evaluation, dapsone was deemed appropriate for the treatment of ICU patients.

**Figure 4:**
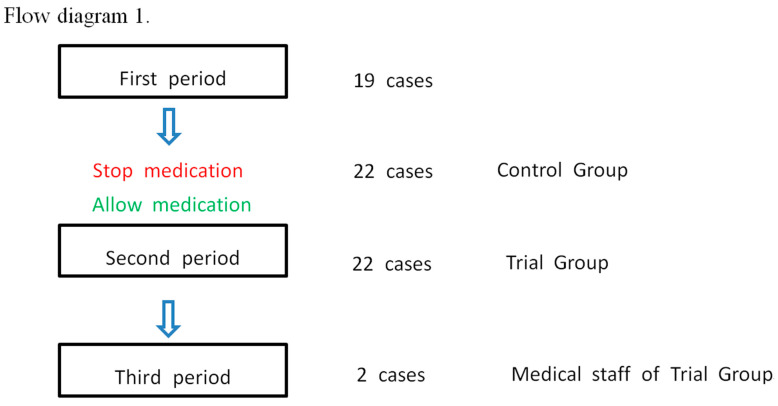
Flow diagram for cross-sectional study. An off-label medication was administered in 19 cases, and a control group (22 cases) and a trial group (22 cases) were observed [14].

The case–control in the ICU was symmetrical (22/22) (Figure 4). The results from the medical treatment from 21 December to 29 December 2020 were statistically analyzed. When decreased FIO_2_ and no further progression of hypoxia were used as criteria for the effectiveness of dapsone, the results were statistically significant according to Fisher’s exact test, and the chi-square test (Supplementary Materials Section 3).

### 4.1. Statistics 1: Chi-Square Statistics

The comparison was made assuming that only the decreased FIO_2_ was influential in the entire dapsone (+) group and dapsone (−) group, which applied to only the ARDS onset stage (Table 4).

### 4.2. Statistics 2: Fisher’s Exact Test

When using the chi-square test, there were cases where zero was entered into the cell, so this was replaced with 1. Fisher’s test was conducted again to compensate for the Chi-squared test (Table 5).

Both results for the dapsone trial group are significant for the ARDS onset stage and ARDS aggravated stage. Moreover, we examined the ARDS onset stage only. There were eight ARDS onset patients who received standard COVID-19 treatment with dapsone (a total of 22 patients). They did not die, except for one patient who did not take dapsone after relapse. Eight of twenty ARDS onset patients (a total of 22 patients) who received standard COVID-19 treatment without dapsone died.

### 4.3. Statistics 3: Chi-Squared Test for ARDS Onset and Mortality

The mortality rates at the ARDS onset stage were 0 and 40%, respectively. The 17 participants at the ARDS onset stage who received the dapsone intervention (M = 1.7, SD = 2.6268) compared to the 20 participants in the control group (M = 2.8, SD = 3.7947) demonstrated a significantly lower mortality score. A chi-square test of independence was performed to examine the mortality relation between ARDS onset (with dapsone) and ARDS onset (without dapsone). The relation between these variables was significant: The chi-square statistic is 5.8108. The *p*-value is 0.015928 (significant at *p* < 0.05). The ARDS onset (with dapsone) group was more likely to survive than the ARDS onset (without dapsone) group (Table 6).

#### Significance Level

Dapsone can be administered to all the ARDS onset patients. The involvement of the brainstem, especially the pre-Bötzinger complex, could explain the respiratory failure and high death rate of the COVID-19 ARDS patients and the sudden recovery of the ARDS onset patients after taking dapsone with standard COVID-19 treatment. It is recommended to use 200 mg of dapsone as the standard dosage at the beginning of ARDS according to clinical experience. Furthermore, even after discharge, to suppress inflammasomes and prevent sequelae, 50–100 mg should be administered daily.

## 5. Treatment Mechanisms

The mode of dapsone–DNA interaction has been demonstrated using biophysical and in silico molecular docking techniques. Various research methods, such as ultraviolet–visible and titrimetric fluorescence studies, circular dichroism, thermal denaturation and viscosity experiments, and competitive binding studies, have shown that dapsone non-covalently binds to the minor groove of DNA [6,48].

The molecular properties of dapsone have been elucidated at the chemical bonding, atomic, and molecular levels, including the electron density and Laplacian delocalization index [49,50]. The oxidation mechanism of dapsone is explained by electron transfer, and its redox properties are dependent on amine and sulfone moieties. The aniline ring has a nucleophilic moiety. It confers biological properties via a redox mechanism, mainly electron transfer or oxidation [51]. The topological properties of dapsone, such as its electron density and Laplacian delocalization index, reveal a very high positive Bader atomic charge (2.36e), as it is attached to the two most electronegative oxygen (O) atoms (−1.27e) on both sides. The electron localization function has been used to visualize and deduce information on the lone pair and the subshells of the electrostatic potential regions susceptible to nucleophilic and electrophilic attack [49]. The negative potential of the vicinity of O and O atoms is vulnerable to severe electrophilic attack. The nucleophilic/electrophilic region of dapsone interacts with amino acids by molecular bonding. Dapsone has a structure that can reduce the production of the sulfur radical by electron charge transfer because it is structurally similar to methionine sulfoxide.

Dapsone has been noted in various diverse neuropathological findings, including those on its mysterious sensory manifestations observed via Heisenberg’s uncertainty principle in quantum mechanics [52]. Dapsone has been used as a therapeutic agent for mild cognitive impairment [9,21], AD [14], PD [22,23], seizures [24], strokes [13,25,26], and, finally, COVID-19 ARDS [6].

When we detected the absorption wavelength values for the electronic spectra of dapsone, the protonated dapsone species were strongly dependent on pH through the excited states. Dapsone is a small molecule with anti-inflammatory and immunosuppressive properties as well as antibacterial and antibiotic properties. Dapsone passes through the BBB [53,54], and high-dose sulfadiazine results in an effective CSF concentration in humans [46].

Mechanism 1: Myeloperoxidase is an oxidoreductase that catalyzes the following chemical reaction: H_2_O_2_ + Cl^−^ = H_2_O + OCl^−^. Dapsone binds to myeloperoxidase and regulates the production of hypochlorite. It reduces the inflammatory responses of cells.

Mechanism 2: The nucleophilic/electrophilic region of dapsone interacts with amino acids by molecular bonding. Neurotoxicity, aggregation, and free radical formation are initiated by the methionine (Met) residue at position 35 in the C-terminal domain of amyloid β peptides (Aβ) [55,56,57]. The two-electron oxidation of bicarbonate is mediated by hydrogen peroxide after the generation of peroxymonocarbonate (HCO4^−^). The bicarbonate/carbon dioxide pair stimulates one-electron oxidation. Carbonate radical anions (CO3^●−^) mediate one-electron reactions to promote one-electron oxidation, efficiently oxidizing the thioether sulfur of the Met residue to sulfur radical cations (MetS^●+^) [58]. Dapsone has a structure that can reduce the production of the positively charged sulfur radical because it has a structure similar to that of methionine sulfoxide.

Mechanism 3: Due to its nucleophilic properties, dapsone competes with ubiquitin (Ub). Before Ub is loaded onto the substrate, the Ub-activating (E1)/Ub-conjugating (E2)/E3 ligase acts at each stage of the ubiquitination process, and the enzymes are all able to carry ubiquitin via a thioester linkage, which allows an energetically favorable attack of the substrate nucleophile. Dapsone can compete with the ubiquitination cascade. An identical mechanism can potentially ubiquitinate cysteine thiols and hydroxyls on serines, threonines, leucines, and tyrosines.

Mechanism 4: Dapsone noncovalently binds/interacts with the minor groove of DNA. The relative energy of the dapsone–DNA interaction/binding is −6.22 kcal mol^−1^, as estimated by in silico studies. The docking analysis further revealed that dapsone preferentially binds to AT-rich regions of DNA [48]. The nucleophilic properties of dapsone mean that it competes with NLRP3. ORF8b activates NLRP3 through direct interaction with the AT-rich repeat domain of NLRP3 [6].

Dapsone has been used to improve the efficiency of newly developed drugs in treating significant diseases.

## 6. Investigation of SARS-CoV-2 Vaccination and Nanobody Drugs

Messenger RNA (mRNA) is a crucial molecule of life in almost all aspects of cell biology, and synthetic mRNA has been engineered to resemble mature and processed mRNA molecules in the cytoplasm of eukaryotic cells [59]. A lipoplex (LPX) protects RNA from extracellular ribonucleases by optimally adjusting the net charge. We can intravenously administer RNA-lipoplexes (RNA-LPXs) as well-known lipid carriers for the systemic delivery of vaccine antigens. In a study, RNA-LPXs encoding viral or mutant neo-antigens or endogenous self-antigens induced potent effector and memory T-cell responses; consequently, IFNα and robust antigen-specific T-cell responses were induced in the first three melanoma patients [60]. An RNA-LPX that encoded the receptor-binding domain (RBD) of the SARS-CoV-2 spike protein was constructed [61], and RBD-binding IgG concentrations and SARS-CoV-2-neutralizing titers in the sera were increased with the dose and after a second dose [61]. A two-dose-regimen vaccine conferred 95% protection against COVID-19 in younger and older persons [62]. However, clinical observations indicate a potential risk of illness after successful vaccination and subsequent infection with the variant virus [63].

Researchers identified nanobodies that specifically bind to the RBD of the virus with an equilibrium dissociation constant between 2 and 22 nmol and neutralized SARS-CoV-2 infection by 50% in a plaque reduction assay at concentrations ranging from 48 to 185 nmol; the results are similar to those achieved with monoclonal antibodies [64]. Nanobodies can be made with the use of prokaryotic expression systems that are easier to manufacture than standard monoclonal antibodies.

Lipid nanoparticles and nanobodies are under clinical investigation for treating various diseases, from cancer to infectious diseases. We highly desire therapies that are safe and effective against variants and offer an alternative to intravenously administered antibody drugs.

The use of oral therapeutics that modulate inflammasomes without compromising the adaptive immune response are likely to be the most effective therapeutic strategy. Here, we suggest that the dapsone–DNA complex can be used as an oral therapeutic for SARS-CoV-2 ARDS. Unlike RNA-RPXs, dapsone does not bind to the RBD but formulates the dapsone–DNA complex, which prevents the exacerbation of SARS-CoV-2 inflammasomes in the brain. Moreover, when RBCs split in the blood microcirculation, they release iron-rich, strongly magnetic nanoparticles associated with diverse SARS-CoV-2 pathology in mitochondria, the endoplasmic reticulum, and the sites of near-contact between the mitochondrion and the endoplasmic reticulum (ER) (mitochondrion–ER), axons, and dendrites [65].

Dapsone inhibits myeloperoxidase (MPO), and the peroxidase activity of heme-bound Aβ is associated with AD [66]. Aβ plays a key role by oxidatively impairing the capacity of red blood cells (RBCs) to deliver oxygen to the brain, and RBC deformability leads to abnormalities in the blood microcirculation [67]. Native MPO is a homodimer with two identical monomeric MPOs connected by a single disulfide bond [68], but hemi-MPO resulting from disulfide cleavage can be produced. Two types of MPO bind to the RBCs’ plasma membranes, which leads to reduced cell resistance to osmotic and acidic hemolysis, a reduction in cell elasticity, and significant changes in cell volume, morphology, and the conductance of ion channels in the RBC’s plasma membrane. MPO, an oxidant-producing enzyme, was shown to cause RBC deformability, leading to abnormalities in the blood microcirculation [69]. Dapsone is a well-known MPO inhibitor [9] that protects neurons from SARS-CoV-2 inflammasomes through RBC splitting and ameliorates SARS-CoV-2 ARDS [27]. Dapsone acts as a competitor of canonical/noncanonical ubiquitylation, NLRP3 inflammasome formation, Higgins’ cascade, and strongly magnetic iron-rich nanoparticles.

Proteins covalently attached to DNA are common and impose physical obstacles to DNA replication, repair, transcription, and recombination [70]. Large DNA–protein crosslinks can be cleaved into DNA–peptide crosslinks, but these smaller fragments also disrupt normal replication [71].

The dapsone–DNA complex may disrupt DNA replication, repair, transcription, and recombination of SARS-CoV-2 inflammasomes based on the fact that it has previously been used as an adjuvant [15], alternative [16], augmentation [17,18], or active ingredient [21,22] by clinicians. Dapsone has been used as a therapeutic agent for mild cognitive impairment [9,21], AD [14], PD [22,23], seizures [24], strokes [13,25,26], and, finally, COVID-19 ARDS [6,27] before dapsone was identified as an inflammasome competitor. This indicates that dapsone can be used along with an oral DNA vaccine for physically obstructing the replication of DNA in inflammasomes.

Recently, incident sequelae have been identified in the respiratory system and several others, including the nervous system, leading to neurocognitive disorders, mental health disorders, metabolic disorders, cardiovascular disorders, gastrointestinal disorders, malaise, fatigue, musculoskeletal pain, and anemia, with the increased incident use of several therapeutics, including pain medications, antidepressants, anxiolytics, antihypertensives, and oral hypoglycemics, and evidence of laboratory abnormalities for multiple organ systems [72]. This also indicates that dapsone can be used to treat sequelae, by the mechanism of physical obstacles to the replication of DNA in inflammasomes exacerbating states, if the doctor prescribes dapsone and monitors its side effects to treat the disease

## 7. Conclusions and Future Directions

Given the existing knowledge of SARS-CoV-2 inflammasomes and sequelae, the wide range of symptoms associated with COVID-19 is not surprising, and sequelae are also likely to be increasingly observed in SARS-CoV-2 survivors. We need to go beyond the traditional vaccine concept and quickly introduce ad hoc prevention and treatment methods. Dapsone is a drug with side effects; however, these are manageable, and, thus, its use is worth exploring.

If doctors prescribe dapsone to patients in the ARDS onset stage, those with COVID-19 may have a higher chance of survival.

## Figures and Tables

**Table 1:** The different neurological manifestations of SARS-CoV-2′s and dapsone’s effects in humans.

Clinical Manifestations	SARS-CoV-2 Symptoms	Dapsone’s Effects	
Hypersensitivity reactions	SARS-CoV-2 symptoms are like a severe idiosyncratic reaction to dapsone characterized by the clinical triad of fever, rash, and systemic involvement, which can cause powerful organ (heart, kidney, lung, and brain) dysfunction [28]	The syndrome is a severe idiosyncratic reaction to dapsone characterized by the clinical triad of fever, rash, and systemic involvement (most commonly of the liver and the hematologic system), which can cause severe organ dysfunction [29]	Similar
Hematology laboratory	Focal fibrin clusters mixed with mononuclear inflammatory cells, decreased eosinophils, decreased lymphocytes, increased neutrophils [30], lymphopenia, leukocytosis, neutrophilia, and thrombocytopenia [31]	Leukocytosis and eosinophilia [32], resembling a mononucleosis infection [33]	Similar
Anemia	Thrombocytopenia and consumptive coagulopathy [31]	Hemolytic anemia and methemoglobinemia [34]	Similar
Liver disease, pancreatic disease	Clinically significant liver injury is uncommon [35]; pancreatic cells highly express ACE2 [36]	Hepatitis/liver toxicity [33]; cholangitis, colitis, and thyroiditis [37]; pancreatitis and pleural effusion [38]	Similar
Renal disease	Severe collapsing, focal segmental glomerulosclerosis, and acute tubular necrosis [39]	Acute renal failure [33]	Similar
Cardiac disease	Acute myocardial injury and chronic damage to the cardiovascular system [40]	Myocarditis, and dapsone-induced hypersensitivity syndrome-associated complete atrioventricular block [37]; myocardial injury [41]	Similar
Pulmonary disease	Coronavirus disease (COVID-19)-related pneumonia [28,40]	Pneumonitis [37]; pneumonia or multiple organ failure [41]	Similar
Neurologic disease	Large-vessel stroke [42] encephalopathy, prominent agitation and confusion, and corticospinal tract signs [43,44]	Recovery of dementia syndrome following treatment of brain inflammation [45]; dapsone alleviated MCI, early AD, and stroke [13]	Different

**Table 2:** AD prevalence (+/−) in the dapsone prescription (+)/non-prescription (−) group.

Year	Dapsone (+)	Dapsone (−)	AD (+) Total	Dapsone (+)	Dapsone (−)	AD (−) Total	Dapsone (+)/AD (+)	Dapsone (+)/AD (−)
2005	18	19	37	290	417	707	0.4865	0.4102
2006	20	37	57	302	363	665	0.3509	0.4541
2007	22	51	73	317	332	649	0.3014	0.4884
2008	22	58	80	310	312	622	0.2750	0.4984
2009	19	66	85	300	283	583	0.2235	0.5146
2010	25	82	107	270	286	556	0.2336	0.4856
2011	35	98	133	255	268	523	0.2632	0.4876
2012	39	135	174	238	241	479	0.2241	0.4969
2013	34	172	206	195	248	443	0.1650	0.4402
2014	25	190	215	172	236	408	0.1163	0.4216
2015	26	242	268	167	168	335	0.0970	0.4985
2016	33	255	288	154	149	303	0.1146	0.5083
2017	37	268	305	143	115	258	0.1213	0.5543
2018	45	292	337	132	87	219	0.1335	0.6027
2019	46	334	380	114	40	154	0.1211	0.7403
2020	32	352	384	109	4	113	0.0833	0.9646

Independent *t*-test (two-tailed): the *t*-value is −7.41861; the *p*-value is < 0.00001; the result is considered significant at *p* < 0.05. Dependent *t*-test (two-tailed): the value of *t* is 6.079808; the value of *p* is 0.00002; the result is significant at *p* < 0.05 (Supplementary Materials Section 1).

**Table 3:**
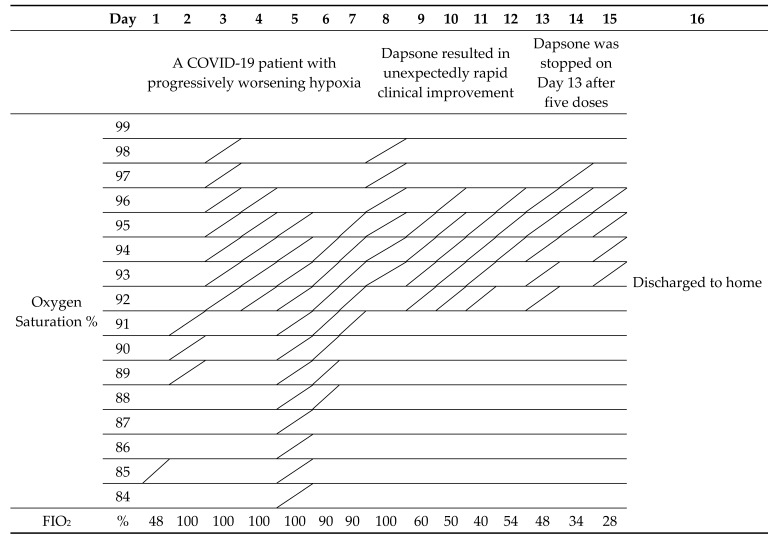
O_2_ saturation graph (Supplementary Materials Section 2, Case 3).

**Table 4:** Chi-squared test.

Study 3	Decreased FIO_2_	Others	Row Totals
Dapsone (+) onset	7 (4.29) (1.72)	1 (3.71) (1.98)	8
Dapsone (−) onset	8 (10.71) (0.69)	12 (9.29) (0.79)	20
Totals	15	13	28

The chi-square statistic is 5.1836. The *p*-value is 0.022801. The result is considered significant at *p* < 0.05.

**Table 5:** Fisher’s exact test calculator.

Study 2–4	decFIO_2_ + No Progressive	Progressive	Row Totals
Dapsone (+) onset + aggravated	17	3	20
Dapsone (+) severe	0	2	2
Total	17	5	22

Fisher’s exact test statistic value is 0.0433. The result is considered significant at *p* < 0.05.

**Table 6:** Chi-squared test for mortality.

	Death	Survival	Row Totals
ARDS onset (with dapsone)	1	16	17
ARDS onset (without dapsone)	8	12	20
Totals	9	28	37

## Data Availability

Data sharing not applicable.

## Supplementary Materials

The following are available online at https://www.mdpi.com/article/10.3390/vaccines9060635/s1.
