# Supplementary material for: Specific Treatment Exists for SARS-CoV-2 ARDS
Source: Vaccines (Basel). 2021 Jun 10;9(6):635. doi: 10.3390/vaccines9060635 (PMC8229893; doi:10.3390/vaccines9060635)
Supplement: Supplementary file 1 [file vaccines-09-00635-s001.zip › vaccines-1243759-supplementary.pdf]

Supplementary material for

Specific Treatment exists for SARS-CoV-2 ARDS

Badar Kanwar, Chul Joong Lee and Jong-Hoon Lee *

내용

Section 1. 3

Supplementary Table 1. AD Prevalence rate in the Dapsone (+) group. 3

T-Test Calculator for 2 Independent Means 4

T-Test Calculator for 2 Independent Means Site 5

T Test Calculator for 2 Dependent Means 6

T Test Calculator for 2 Dependent Means Site 6

T-Test Calculator for 2 Independent Means: Mortality 7

Chi-squared test for MORTALITY 8

Section 2. Case Reports. 9

Case 1 9

Case 2 9

Case 3 9

Case 4 10

Case 5 11

Section 3. 12

Patient management report 12

Statistics 1. The chi-square statistics 14

Statistics 2. Fischer's exact test 15

Section 1.

Supplementary Table 1. AD Prevalence rate in the Dapsone (+) group.

Year	Dapsone (+)	Dapsone (-)	AD (+) Total	Dapsone (+)	Dapsone (-)	AD (-) Total	Rate:AD (+) Dapsone (+)	Rate:AD (-) Dapsone (+)
**2005**	18	19	37	290	417	707	0.4865	0.4102
**2006**	20	37	57	302	363	665	0.3509	0.4541
**2007**	22	51	73	317	332	649	0.3014	0.4884
**2008**	22	58	80	310	312	622	0.2750	0.4984
**2009**	19	66	85	300	283	583	0.2235	0.5146
**2010**	25	82	107	270	286	556	0.2336	0.4856
**2011**	35	98	133	255	268	523	0.2632	0.4876
**2012**	39	135	174	238	241	479	0.2241	0.4969
**2013**	34	172	206	195	248	443	0.1650	0.4402
**2014**	25	190	215	172	236	408	0.1163	0.4216
**2015**	26	242	268	167	168	335	0.0970	0.4985
**2016**	33	255	288	154	149	303	0.1146	0.5083
**2017**	37	268	305	143	115	258	0.1213	0.5543
**2018**	45	292	337	132	87	219	0.1335	0.6027
**2019**	46	334	380	114	40	154	0.1211	0.7403
**2020**	32	352	384	109	4	113	0.0833	0.9646

Independent T-Test (two-tailed): The t-value is -7.41861. The p-value is < .00001. The result is significant at p < .05. Dependent T-Test (two-tailed): The value of t is 6.079808. The value of p is .00002. The result is significant at p < .05.

T-Test Calculator for 2 Independent Means

T-Test Calculator for 2 Independent Means

Success!

Explanation of results

The output of this calculator is pretty straightforward. The values of *t* and *p* appear at the bottom of the page. If the text is blue, your result is significant; if it's red, it's not. The only thing that might catch you out is the way that we've rounded the data. The data you see in front of you, apart from the *t* and *p* values at the page bottom, has been rounded to 2 significant figures. However, we did not round when actually calculating the values of *t* and *p*. This means if you try to calculate these values on the basis of the summary data provided here, you're likely going to end up with a different, less accurate, result. This is especially the case if you're dealing with numbers that are fractions of 1.

Treatment 1 (*X*)	*Diff* (*X* - *M*)	*Sq. Diff* $(X - M)^2$
0.4865	0.28	0.08
0.3509	0.14	0.02
0.3014	0.09	0.01
0.2750	0.07	0.00
0.2235	0.02	0.00
0.2336	0.03	0.00
0.2632	0.06	0.00
0.2241	0.02	0.00
0.1650	-0.04	0.00
0.1163	-0.09	0.01
0.0970	-0.11	0.01
0.1146	-0.09	0.01
0.1213	-0.09	0.01
0.1335	-0.07	0.01
0.1211	-0.09	0.01
0.0833	-0.12	0.02
	M: 0.21	SS: 0.18

Treatment 2 (*X*)	*Diff* (*X* - *M*)	*Sq. Diff* $(X - M)^2$
0.4102	-0.13	0.02
0.4541	-0.08	0.01
0.4884	-0.05	0.00
0.4984	-0.04	0.00
0.5146	-0.02	0.00
0.4856	-0.05	0.00
0.4876	-0.05	0.00
0.4969	-0.04	0.00
0.4402	-0.10	0.01
0.4216	-0.11	0.01
0.4985	-0.04	0.00
0.5083	-0.03	0.00
0.5543	0.02	0.00
0.6027	0.07	0.00
0.7403	0.20	0.04
0.9646	0.43	0.18
	M: 0.54	SS: 0.29

Significance Level:

- ○ .01
- ◉ .05
- ○ .10

One-tailed or two-tailed hypothesis?:

- ○ One-tailed
- ◉ Two-tailed

Difference Scores Calculations

Treatment 1

N_1: 16
$df_1 = N - 1 = 16 - 1 = 15$
M_1: 0.21
SS_1: 0.18
$s^2_1 = SS_1/(N - 1) = 0.18/(16-1) = 0.01$

Treatment 2

N_2: 16
$df_2 = N - 1 = 16 - 1 = 15$
M_2: 0.54
SS_2: 0.29
$s^2_2 = SS_2/(N - 1) = 0.29/(16-1) = 0.02$

T-value Calculation

$s^2_p = ((df_1/(df_1 + df_2)) * s^2_1) + ((df_2/(df_2 + df_2)) * s^2_2) = ((15/30) * 0.01) + ((15/30) * 0.02) = 0.02$

$s^2_{M_1} = s^2_p/N_1 = 0.02/16 = 0$
$s^2_{M_2} = s^2_p/N_2 = 0.02/16 = 0$

$t = (M_1 - M_2)/\sqrt{(s^2_{M_1} + s^2_{M_2})} = -0.33/\sqrt{0} = -7.42$

The t-value is -7.41861. The p-value is < .00001. The result is significant at $p < .05$.

Note: If you wish to calculate the effect size, this calculator will do the job.

T-Test Calculator for 2 Independent Means Site

https://www.socscistatistics.com/tests/studentttest/default.aspx

T Test Calculator for 2 Dependent Means

T-Test Calculator for 2 Dependent Means

The value of *t* is 6.079808.

Explanation of results

The output of this calculator is pretty straightforward. The values of *t* and *p* appear at the bottom of the page. If the text is blue, your result is significant; if it's red, it's not. The only thing that might catch you out is the way that we've rounded the data. The data you see in front of you, apart from the *t* and *p* values, has been rounded to 2 significant figures. However, we did not round when actually calculating the values of *t* and *p*. This means that if you try to calculate these values on the basis of the summary data provided here, you're likely going to end up with a slightly different - and less accurate - result.

Treatment 1	Treatment 2	*Diff* (T2 - T1)	*Dev* (Diff - M)	*Sq. Dev*
0.4865	0.4102	-0.08	-0.4	0.16
0.3509	0.4541	0.1	-0.23	0.05
0.3014	0.4884	0.19	-0.14	0.02
0.2750	0.4984	0.22	-0.11	0.01
0.2235	0.5146	0.29	-0.04	0
0.2336	0.4856	0.25	-0.08	0.01
0.2632	0.4876	0.22	-0.1	0.01
0.2241	0.4969	0.27	-0.06	0
0.1650	0.4402	0.28	-0.05	0
0.1163	0.4216	0.31	-0.02	0
0.0970	0.4985	0.4	0.07	0.01
0.1146	0.5083	0.39	0.07	0
0.1213	0.5543	0.43	0.1	0.01
0.1335	0.6027	0.47	0.14	0.02
0.1211	0.7403	0.62	0.29	0.08
0.0833	0.9646	0.88	0.55	0.31
		M: 0.33		S: 0.7

Significance Level:

- ○ 0.01
- ◉ 0.05
- ○ 0.10

One-tailed or two-tailed hypothesis?:

- ○ One-tailed
- ◉ Two-tailed

<u>Difference Scores Calculations</u>

Mean: 0.33

$\mu = 0$

$S^2 = SS/df = 0.7/(16-1) = 0.05$

$S^2_M = S^2/N = 0.05/16 = 0$

$S_M = \sqrt{S^2_M} = \sqrt{0} = 0.05$

<u>T-value Calculation</u>

$t = (M - \mu)/S_M = (0.33 - 0)/0.05 = 6.08$

The value of *t* is 6.079808. The value of *p* is .00002. The result is significant at p < .05.

T Test Calculator for 2 Dependent Means Site

https://www.socscistatistics.com/tests/ttestdependent/default.aspx

T-Test Calculator for 2 Independent Means: Mortality

Mortality at the ARDS-Onset stage from Hunt Regional Hospital in Greenville, TX

	Decrease in FIO_2			Progression of hypoxia			No further progression of hypoxia			Mortality
Period	1st	2nd	3rd	1st	2nd	3rd	1st	2nd	3rd	
ARDS onset (death)	*6*	7	2	0	0	0	1	1	0	0/17
ARDS onset (death)	*0*	8	0	0	8 (8)	0	0	4	0	8/20

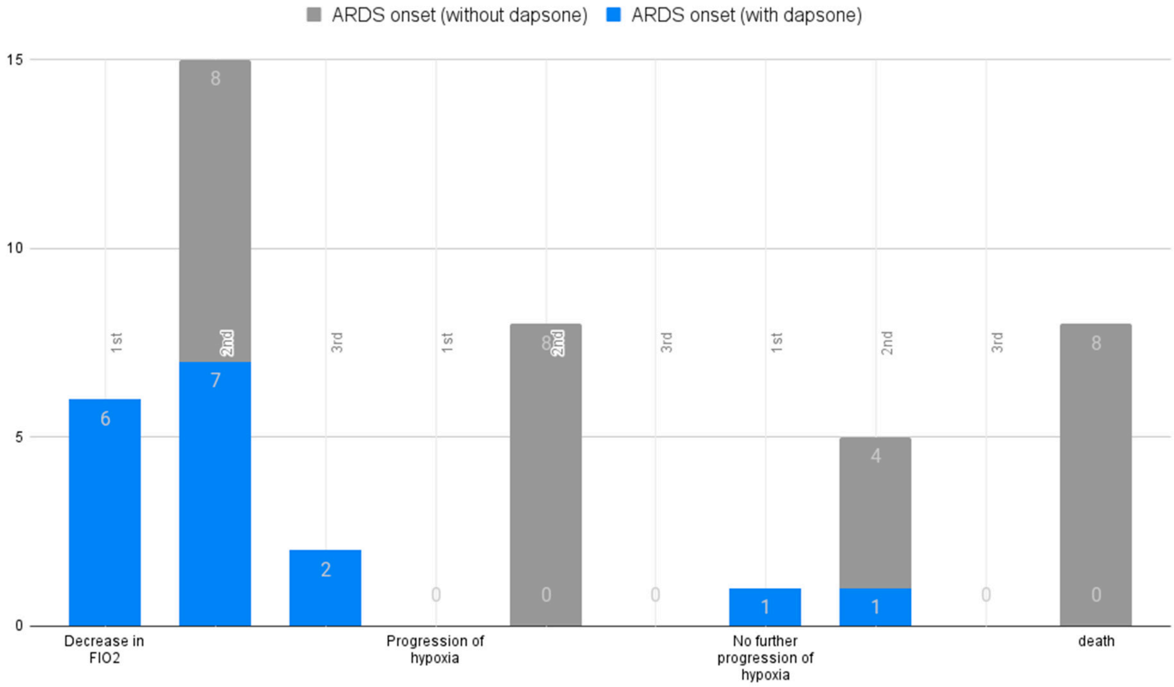

Chi-squared test for MORTALITY

	DEATH	SURVIVAL	*Marginal Row Totals*
**ARDS onset (with dapsone)**	1 (4.14) [2.38]	16 (12.86) [0.76]	17
**ARDS onset (without dapsone)**	8 (4.86) [2.02]	12 (15.14) [0.65]	20
Marginal Column Totals	9	28	37 (Grand Total)

The chi-square statistic is 5.8108. The p-value is .015928. Significant at $p < .05$.

The chi-square statistic with Yates correction is 4.1052. The p-value is .042752. Significant at $p < .05$.

Section 2. Case Reports.

Case 1

65 y/o female with a history of hypertension, breast cancer in remission, and peripheral vascular disease. She was admitted for COVID-19 pneumonia, meds on admission, O_2 sat, p, r, bp, temp. Convalescent plasma, ceftriaxone, doxycycline and dexamethasone were started with improvement and discharged on room air after nine days. Two days after discharge, she returned with worsening dyspnea, productive cough, 36.8 °C, O_2 sat 83%, p 91, r 18. She was hypoxic and started on 3L/min. Bilateral infiltrates, and deteriorating status necessitated transfer to ICU on day 4. She required BiPAP with 100% O_2 and our standard COVID-19 therapy of, dexamethasone, ascorbic acid, zinc sulfate, and remdesivir, and convalescent plasma with cefepime.

After progressive dyspnea and worsening desaturation, a pattern we see portending fatal outcome, with no further standard treatments to offer, we started oral Dapsone 100 mg on hospital day 4. Seventy-two hours after starting Dapsone, O_2 requirement was 80% O_2 sat was 91%..p 92.r 20. On hospital day 13, dapsone dose was increased to 200 mg PO daily. 24 hours later O_2 sat was 92%, r 19 ..p 82...on 75% O_2. At 48 after dapsone increase, O_2 was 65%...r 22..p 88. The patient was discharged from the ICU to a general medical ward with complete full resolution.

Case 2

71 y/o male with a history of Type II diabetes mellitus, hypertension, coronary artery disease, and obesity presented to ER on Nov 7, 2020, with worsening dyspnea, cough, and fatigue for one week. His wife was diagnosed with COVID 10 days previously. After admission, with bilateral infiltrates on the chest film, and O_2 sat. 90%, 2L/min O_2 was started. The temperature was 37.1 °C, BP 140/71, and respirations 21/min. COVID-19 was PCR positive. In the attempt to reverse the progressing dyspnea with increasing O_2 Dapsone 100 mg PO daily was started with standard COVID-19 therapy. One day later, O_2 requirement went up to 10L/min. Dapsone dose was increased to 200 mg/day. On 11/17, the patient was symptomatically better, and O_2 requirement decreased to 8 L/min, O_2 sat was increased. On discharge on hospital day, discharge meds were prescribed.

Case 3

A 62 y/old female with well-treated hypothyroidism, presented to the emergency room with a several day history of progressively worsening chills, non-productive cough, dyspnea and malaise. Several days prior to admission her family doctor started her on amoxicillin/clavulanate and methylprednisolone after diagnosing COVID19 based on a positive PCR test. BP was 110/54, P 59, R 18, and temperature 36.7 C. On admission to hospital, oxygen saturation was 85% with 8 L/min 100% O_2 by nasal cannula (NC). She was started on enoxaparin, ascorbic acid 1,000 mg twice daily, Vitamin D3 1,000 units p.o. twice

daily, and dexamethasone 4 mg IV every 12 hours. Azithromycin 500 mg IV daily and ceftriaxone 2 G IV daily was added after her CT chest showed bilateral extensive infiltrates. WBC were 6.6, C-reactive protein 147 [normal <10]. She remained afebrile.

Remidesivir and convalescent plasma were added on hospital day 3. She was transferred to ICU due to increasing FiO_2 requirements. See the Table for an overview of her changing oxygenation status. In ICU she was started on BiPAP 15/5 cm H_2O, FiO_2 90%, alternating with high flow nasal cannula (HFNC) O_2 50 L/min and FiO2 95%. O2 saturation varied from 88 to 94%.

On day 8, O2 saturation was 89% on above medicines and ventilation support. She presented a deteriorating picture that we commonly see in COVID19 cases that do not end well. After thorough discussion of the risks and unproven value of dapsone, this was added at 100 mg p.o. daily. Twenty-four hours later, FiO_2 requirement decreased to HFNC 50 L/min and FiO_2 80%, with O_2 saturation 94-97% allowing decrease of FiO_2 to 50%.

On day 11, the fourth day of dapsone, patient was transferred out of ICU on FiO_2 8 L/min NC, O_2 saturation 93-96%, P 69, R17. Dapsone was stopped on day 13 after five doses. On day 15 she was discharged to home on 2 L/min via NC with temperature 36.8 C, P 78, BP 120/77, R 19. Her C reactive protein 2 days prior to discharge was 0.5. Her discharge medications were ascorbic acid 1,000 mg twice daily, vitamin D3 5,000 units daily, dexamethasone 6 mg daily on a taper schedule.

Case 4

59 years old healthy Hispanic female without any medical problems. She presented on 11/11/202 to our ER after being diagnosed 11 days ago at another facility with COVID-19. She was experiencing worsening shortness of breath, and her room air pulse Ox was in the 50% range. Her Heart rate, 97/min, BP, 137/57, RR 30/min, and temperature 37.3 °C.

She was started on HFNC at 50LMP flow and 100% FIO_2 and admitted to the intensive care unit. CT chest revealed no PE, bilateral ground-glass opacities. She initiated our standard combo medicine for COVID. The patient was desaturating on HFNC into low 80s and high 70s but without apparent distress. On 11/12/2020, she was offered Dapsone, but she declined. However, it was started on 11/13/2020 at 200 mg daily following internal discussion on compassionate grounds. The patient remained stable on HFNC 50L/min and 100% FIO_2 during the day and BiPAP with 100% FIO_2 during the night until 11/24/2020 with SaO_2 88-92%. On 11/24/2020 patient appeared cyanotic. Methemoglobin was checked, and it was 11.2. The patient started Cimetidine 400 mg PO TID. On 11/25/2020, her Dapsone dose was increased to 300 mg daily. Due to worsening hypoxia, chest CT was done repeatedly. It revealed bilateral PE with RV strain. The patient has currently treated with TPA 100 mg.

Case 5

67 y/o male with a history of hypertension, testicular carcinoma in remission was admitted with a COVID-19 diagnosis and treated with the standard Covid-19 treatment. After six days he was discharged to home on O2% sat, and discharge meds. Post-discharge he was readmitted with dyspnea, non-productive cough and fatigue, O2sat 80% BP 152/78, p 91/min, R 18/min and temperature 37.1 °C. Bilateral ground-glass opacities with right lower lung pulmonary embolism was diagnosed. 15L/min O2 was inadequate, necessitating intubation with 100% O2 by high-frequency mech vent He was treated with enoxaparin. The patient was later moved to BiPAP with 100% FIO2. On 11/02/202, due to worsening Hypoxia despite BiPAP and respiratory distress patient was intubated. After intubated patient required 75-100% O2 and PEEP 12-15 cm of H2O to maintain SaO2 in low 90s, his course was complicated by GI bleed and renal failure.

0n 11/11/2020, Dapsone was initiated at 100 mg daily dose. There was no meaningful improvement observed in the FIO2 requirement. The patient expired on 11/15/2020.

Section 3.

The Ethics and Administrative Committee of Hunt Regional Hospital in Greenville, TX, approved this clinical treatment based on the World Medical Association's Declaration of Helsinki. All patients (or their parents or guardians) provided written informed consent.

The criterion for ARDS onset was the requirement of FIO2 via simple nasal cannulation of up to 15 L/min. The criteria for aggravated cases of ARDS were FiO2 administered via an HFNC (high-flow nasal cannula) of 95-100% and/or bilevel positive airway pressure (BiPAP). The criterion for severe ARDS was the need for mechanical ventilation.

Protocol for administration: Written informed consent should be obtained, and potential side effects should be explained. The common side effects are haemolytic anaemia (in patients with G6PD deficiency)[10], methemoglobinemia, and allergic reaction. The patients should also be informed that currently, G6PD is a send-out test and can take up to 5-7 days. Cimetidine 400 mg orally TID will now be administered to counter dapsone methemoglobinemia side effects[11]. The venous methemoglobin level should be checked every day, and a mild methemoglobin level of 2-10%[12] is well tolerated. Dapsone should be discontinued if the level reaches 15 or above.

Patient management report

1. A total of 22 patients were treated with standard COVID-19 therapy (with dapsone)

**Categories**	**Decrease in FIO_2**	**Progression of hypoxia**	**No further progression of hypoxia**
Onset<br>(FIO_2 requirement	7/22	0/22 (no deaths)	1/22

via simple nasal cannulation of up to 15 L/min)	6/22	3/22 (all deaths)	3/22
Aggravated (FIO$_2$ administered via an HFNC of 95-100% and/or BiPAP)	0/22	2/22 (both deaths)	0/22
Severe (requiring mechanical ventilation)			
**Total**	**13**	**5**	**4**

2. A total of 22 patients treated with standard COVID-19 therapy (without dapsone)

**Categories**	**Decrease in FIO2**	**Progression of hypoxia**	**No further progression of hypoxia**
Onset (FIO2 requirement via simple nasal cannulation of up to 15 L/min)	8/22	8/22 (all deaths)	4/22
	1/22	1/22	0/22

Aggravated (FIO2 administered via an HFNC of 95-100% and/or BiPAP)<br><br>Severe (requiring mechanical ventilation)	0/22	0/22	0/22
**Total**	**9**	**9**	**4**

Statistics 1. The chi-square statistics

1.3. The comparison was made assuming that only the case of decreased FIO_2 was influential in the entire dapsone (+) group and dapsone (-) group, which was applicable to only the ARDS onset stage.

Study 3	dec FIO_2	others	***Row Totals***
Dapsone (+) onset	7 (4.29) [1.72]	1 (3.71) [1.98]	8
Dapsone (-) onset	8 (10.71) [0.69]	12 (9.29) [0.79]	20
Column Totals	15	13	**28 (Total)**

The chi-square statistic is 5.1836. The p-value is .022801. The result is significant at $p < .05$.

Statistics 2. Fischer's exact test

When using the chi-square test, there were cases where 0 was entered into the cell, so this was replaced with 1. Fisher’s test was again conducted to compensate Statistics 1.

Study 2-4	decFIO2+no progress	progression	Marginal Row Totals
Dapone (+) onset + aggrav	17	3	20
Dapone (+) severe	0	2	2
	17	5	22 (Grand Total)

The Fisher exact test statistic value is 0.0433. The result is significant at $p < .05$

With the chi-square test (Studies 2-4), on the basis of decreased FIO2 and no further progression of hypoxia, the addition of dapsone to standard COVID-19 treatment was more effective in ARDS-onset cases than in severe cases.

The 22 patients who received dapsone were divided into onset and aggravated (onset + aggravated) and severe (severe) groups. With Fisher’s exact test, on the basis of decreased FIO2 and no further progression of hypoxia, dapsone was useful, and the results were statistically significant.

Additionally, when the dapsone-treated group was compared with the ARDS onset group and the other groups, there was no meaningful result regardless of whether the criterion for determining the effect was only decreased FIO2 or included no further progression of hypoxia.

Finally, to judge whether the effects of this dapsone treatment (name: **Soon-Joe treatment**) are meaningful, it is more appropriate to conduct Fischer’s exact test than the chi-square test. Of course, the statistic results were the same.
